# Genome-Wide Characterization and Light-Responsive Expression Patterns of B-Box Transcription Factors in *Artemisia argyi*

**DOI:** 10.3390/plants15132003

**Published:** 2026-06-28

**Authors:** Qianwen Zhang, Yuhuan Miao, Sainan Peng, Wunian Feng, Yun Yang, Dahui Liu

**Affiliations:** 1 School of Pharmacy, Hubei University of Chinese Medicine, Wuhan 430065, China; 3395@hbucm.edu.cn (Q.Z.); miaoyh@hbucm.edu.cn (Y.M.); pengsainan0905@stmail.hbucm.edu.cn (S.P.); 2431800794@stmail.hbucm.edu.cn (W.F.); 2431800806@stmail.hbucm.edu.cn (Y.Y.); 2 Hubei Shizhen Laboratory, Hubei University of Chinese Medicine, Wuhan 430065, China

**Keywords:** *Artemisia argyi*, B-box, gene expression, bioinformatics analysis, gene family

## Abstract

For over 3000 years, the perennial herb mugwort (*Artemisia argyi*) has served as a cornerstone of traditional Asian medicine. Its clinical efficacy is driven by a diverse array of specialized metabolites, most notably flavonoids and volatile oils. While B-box (BBX) transcription factors are known to dictate photomorphogenic development and secondary metabolic pathways in plants, this specific gene family has not yet been systematically analyzed in *A. argyi*. Leveraging a chromosome-level genomic assembly, we comprehensively identified and analyzed the complete repertoire of *AarBBX* genes, profiling their structural organization, physicochemical attributes, conserved motifs, promoter architecture, and spatial expression dynamics. The *AarBBX* family segregates into five distinct evolutionary clades and comprises 114 members, exceeding the gene counts in the diploid relatives *Artemisia annua* (27) and *Arabidopsis thaliana* (32), a numerical increase potentially attributable to the tetraploid genome architecture of *A. argyi*. Promoter scanning revealed a high density of *cis*-acting elements linked to light perception and environmental stress responses. Integrating RNA-seq transcriptomics with tissue-specific expression profiling, we identified prominent candidate light-responsive *AarBBX* genes that are highly active in green, photosynthetic tissues and acutely responsive to shifts in light conditions, providing a foundation for future exploration of their potential relationship with secondary metabolic pathways, including flavonoid and terpenoid biosynthesis. Furthermore, we validated the potential operational compartments and structural interactions of these proteins utilizing green fluorescent protein (GFP) subcellular localization and yeast two-hybrid (Y2H) screenings. Collectively, these findings provide new insights into the evolutionary trajectory and regulatory potential of the B-box (BBX) proteins in *A. argyi*, offering a prioritized candidate gene set for subsequent investigations into their potential roles in light-regulated secondary metabolism, including flavonoid and terpenoid pathways.

## 1. Introduction

B-box (BBX) proteins represent a dedicated class of zinc-finger transcription factors distinguished by one or more highly conserved B-box domains. Pervasive across plant lineages, these regulatory proteins modulate a broad spectrum of physiological events, spanning light-mediated development (photomorphogenesis), structural growth, and environmental stress adaptation. In the model plant *Arabidopsis thaliana*, 32 distinct BBX members have been categorized into five structural classes (Groups I through V) based on their specific domain arrangements [1]. Members of Groups I and II are characterized by tandem B-box domains paired with a C-terminal CCT (CONSTANS, CONSTANS-like, and TIMING OF CAB1) motif. Group III proteins feature a single B-box coupled to a CCT domain. Conversely, Groups IV and V contain either one or two B-box domains but completely lack the CCT domain. The B-box structural motif serves as a critical platform for protein–protein interactions, enabling BBX factors to dimerize with family peers or form complexes with heterologous transcriptional modulators to dictate downstream gene activity [2,3,4,5]. A classic example of this is AtBBX32, which physically interacts with AtBBX4 and AtBBX21 via these precise B-box-interfaces [6,7].

BBX proteins also bridge light perception networks with hormone signaling by physically docking with key photomorphogenic effectors like HY5 (ELONGATED HYPOCOTYL 5) and HYH (HY5 HOMOLOG), as well as hormone-responsive components such as ABI5 (ABA Insensitive 5) [2,3].

The B-box domain itself is capable of driving the transcriptional activation of target loci [8,9,10]. The CCT domain, when present, assists in stabilizing protein complexes, guiding transcriptional function, and orchestrating nuclear translocation [11,12,13,14]. Nuclear localization signals embedded directly within the CCT framework ensure that these transcription factors are imported into the nucleus, a step mandatory for their regulatory function [15].

The BBX family occupies a pivotal position in plant light signaling networks, integrating photoreceptor signals with transcriptional responses. By interacting with core machinery components such as COP1 (CONSTITUTIVELY PHOTOMORPHOGENIC 1), HY5, and PIFs (PHYTOCHROME INTERACTING FACTORS), BBX proteins tightly govern developmental transitions, shade avoidance responses, and photoperiod-driven flowering schedules [16].

Although initially characterized in model systems, BBX proteins have recently been shown to play equally critical roles in regulating secondary metabolism (anthocyanins, carotenoids, flavonoids, and terpenoids) in several plant species, including tomato (*Solanum lycopersicum*), eggplant (*Solanum melongena*), pear (*Pyrus pyrifolia*), lettuce (*Lactuca sativa)*, and citrus (*Citrus* spp.), by directly activating key biosynthetic genes such as *PSY1, CHS*, and *F3H*, or by forming regulatory complexes with factors such as HY5 and MYB proteins [8,17,18,19,20,21,22,23,24]. Crucially, within the Asteraceae family, AaBBX22 operates as a positive regulator of artemisinin production in *Artemisia annua*; it directly binds and activates the promoters of pivotal pathway genes, namely *AaADS*, *AaCYP71AV1*, *AaDBR2*, and *AaALDH1*, resulting in substantial artemisinin enrichment in transgenic lines [25]. Furthermore, AaBBX21 coordinates with AaHY5 to stimulate key regulatory nodes of the artemisinin pathway (*AaGSW1*, *AaORA*, and *AaMYB108*), confirming that BBX factors exert direct, precise control over high-value pharmaceutical pathways [19].

Mugwort (*A. argyi*) is a prominent perennial member of the *Artemisia* genus (Asteraceae) with an established history in East Asian medical traditions extending back over 3000 years. Its clinical utility depends heavily on an extensive pool of specialized metabolites, including volatile oils, flavonoids, terpenoids, and phenolic acids. The essential oil fraction is heavily enriched with monoterpenoids and sesquiterpenoids, such as 1,8-cineole, camphor, and borneol, which exhibit potent antimicrobial, antiviral, and bronchodilatory activities. Meanwhile, its major flavonoids, including eupatilin and jaceosidin, display profound anti-inflammatory, antioxidant, and antitumor properties [26,27]. Although the accumulation of these therapeutic compounds varies based on genetic lines, developmental maturity, and microclimatic stresses, the overarching transcriptional circuits that govern these pathways have not yet been fully worked out.

The genomic landscape of *A. argyi* is remarkably large and complex, which has traditionally hindered forward and reverse genetic strategies. Recent chromosome-level assemblies have clarified its evolutionary history and revealed that *A. argyi* (2n = 34) is an allopolyploid that experienced multiple rounds of whole-genome duplication (WGD) [28,29,30]. Its massive ~7.84–8.03 Gb genome comprises more than 73% repetitive sequences and encodes an estimated 279,294 genes, ranking it among the highest gene densities documented within the Asteraceae family [28,29,30]. This complex genomic context underpins the extensive genetic diversity of *A. argyi* and may have further driven the diversification of secondary metabolic pathways via the expansion and functional differentiation of relevant transcription factor families.

The rapid release of high-quality *A. argyi* reference genomes has made genome-wide identification and functional analysis an important approach for elucidating the regulatory mechanisms underlying the biosynthesis of medicinal compounds. Several transcription factor gene families have recently been characterized in *A. argyi*, including MYB and bHLH (basic helix–loop–helix) [31,32]. However, the *AarBBX* gene family, a regulator of light signaling and secondary metabolism, remains uncharacterized.

Deploying a high-quality, chromosome-level genomic assembly [28], we successfully mapped 114 *AarBBX* loci within the *A. argyi* genome. For each identified gene, we systematically analyzed evolutionary lineages, exon–intron architectures, conserved structural motifs, and physical arrangements across chromosomes. We tracked specific gene duplication mechanisms and assessed transcriptional activity under varying light regimes and across diverse plant tissues utilizing a combination of RNA-seq datasets and quantitative real-time PCR (RT-qPCR) validation. To isolate specific candidate factors driving light-mediated specialized metabolism, we conducted targeted subcellular localization assays and protein–protein interaction screens. This comprehensive analysis of the *AarBBX* family within a polyploid medicinal species clarifies the evolutionary pressures shaping these genes. Although this work does not establish direct regulatory roles in secondary metabolism, the prioritized *AarBBX* candidates offer a foundation for future studies that will explore their potential relationship with volatile oil and flavonoid biosynthesis, contingent upon metabolite profiling and genetic validation.

## 2. Materials and Methods

### 2.1. Identification of the AarBBX Transcription Factor Family

#### 2.1.1. Gene Retrieval and Screening

To map the *AarBBX* family, *Arabidopsis* BBX protein sequences were retrieved from TAIR (https://www.arabidopsis.org/index.jsp (accessed on 6 January 2025)) and used as queries for BLAST searches against the *A. argyi* genome (from published data; ref. [28]) via TBtools-II software (v2.148; ref. [33]). All 32 *Arabidopsis thaliana* BBX protein sequences were used as queries for BLASTp searches against the local *A. argyi* protein database, with an e-value cutoff of ≤1 × 10^−5^ and a query coverage ≥50.0%. Concurrently, advanced HMMER searches were performed using the built-in Pfam domain profile for the B-box domain (PF00643), with an e-value threshold of ≤1 × 10^−3^. Only sequences identified by both BLASTp and HMMER (intersection) were retained as candidate BBX homologs, yielding an initial pool of 118 candidate genes. Notably, CD-HIT was intentionally omitted from the pipeline. As this study aims to characterize the complete *BBX* gene family, paralogous genes must be retained as distinct family members. Furthermore, the *A. argyi* genome annotation provided one representative protein sequence per gene locus, so no additional filtering of redundant isoforms or splice variants was required. All candidate sequences were subsequently subjected to manual verification using Pfam (v35.0), SMART (http://smart.embl-heidelberg.de/ (accessed on 12 January 2025)), InterProScan (v5.52-86.0), and NCBI Batch CD-Search (https://www.ncbi.nlm.nih.gov/Structure/bwrpsb/bwrpsb.cgi (accessed on 12 January 2025)) [34,35]. Sequences were excluded if they: (i) lacked at least one intact B-box domain; (ii) possessed an incomplete open reading frame; or (iii) contained additional non-BBX domains and exhibited protein lengths substantially exceeding the typical range of the BBX family (suggesting potential annotation artifacts or fusion proteins), as determined by comparison with the length distribution of validated BBX proteins in *A. thaliana* and *A. annua*. The remaining sequences were designated as *AarBBX* genes. After removing sequences lacking core characteristics, we identified 114 authentic *AarBBX* genes.

#### 2.1.2. Protein Properties

For the 114 AarBBX proteins, we computed physicochemical parameters using ExPASy (https://web.expasy.org/compute_pi/ (accessed on 6 February 2025)): amino acid length, molecular weight, theoretical isoelectric point (pI), aromaticity index, instability index, aliphatic index, and grand average of hydropathicity (GRAVY).

### 2.2. Phylogenetic Analysis

We downloaded 32 AtBBX protein sequences from TAIR. Phylogenetic analysis was performed using the full-length amino acid sequences of BBX proteins from *Artemisia argyi* (114) and *Arabidopsis thaliana* (32). Multiple sequence alignment was conducted using Muscle (integrated in TBtools-II “One Step Build a ML Tree” module) with default parameters. The alignment was automatically trimmed using trimAl (automated1 mode) to remove poorly aligned regions. The best-fit amino acid substitution model was determined using ModelFinder implemented in IQ-TREE (called internally by TBtools-II), and the JTT+R8 model was selected according to the Bayesian Information Criterion (BIC). Maximum likelihood (ML) phylogenetic trees were reconstructed using IQ-TREE under the selected JTT+R8 model with 1000 bootstrap replicates. The tree was visualized and annotated using iTOL (https://itol.embl.de/ (accessed on 6 February 2025)).

Subfamily classification was determined through an integrated approach. The well-characterized subfamily nomenclature of *A. thaliana* BBX proteins served as the primary reference framework, adhering to the established conventions in plant gene family studies. Members of the *A. argyi* BBX subfamily were assigned to corresponding subfamilies based on: (i) their phylogenetic position in the combined maximum likelihood tree reconstructed from the datasets of *A. argyi* and *A. thaliana*, and (ii) their sequence similarity to the *A. thaliana* counterparts. We note that the maximum likelihood (ML) tree revealed some topological differences from earlier neighbor-joining analyses, particularly for subfamilies II and V, where members of *A. thaliana* were not recovered as monophyletic clades. Such discrepancies reflect the greater statistical robustness of ML in addressing sequence divergence and rate heterogeneity. In instances where *A. thaliana* reference members of a given subfamily were not monophyletic in the ML tree, genes from *A. argyi* were assigned to the corresponding subfamily based on their closest phylogenetic affinity and highest sequence similarity to individual members of the *A. thaliana* subfamily, thereby ensuring consistency with the established classification system.

### 2.3. Sequence Characterization

Subcellular localization was characterized using WoLFPSOR (https://wolfpsort.hgc.jp/ (accessed on 6 February 2025)). Conserved domains were identified with NCBI CD-Search, and protein motifs were detected using MEME (https://meme-suite.org/meme/doc/meme.html (accessed on 30 January 2025)) with a search limit of 10 motifs. Phylogenetic relationships, gene structure, domains, and motifs were integrated and visualized in TBtools-II software (v2.154).

### 2.4. Chromosomal Mapping, Collinearity Analysis, and Synteny Analysis

Chromosomal locations of *AarBBX* genes were mapped using TBtools-II software (v2.154). Syntenic relationships within *A. argyi* and between *A. argyi* and *Arabidopsis* (*AtBBX*) or *A. annua* (*AaBBX*) were examined using MCScanX in TBtools-II software (v2.154) [33].

### 2.5. Plant Materials and Treatments

We used ‘Xiang Ai’, a flavonoid-rich *Artemisia argyi* accession from Qichun, Hubei (the source material for genome sequencing), as the experimental subject.

For the tissue-specific sampling for RT-qPCR, Roots (R), rhizomes (Rh), stems (S), and leaf samples at three developmental stages: leaf buds (5 days, L1), young leaves (15 days, L2), and mature leaves (30 days, L3) were collected from *Artemisia argyi* ‘Xiang Ai’ plants. The sampling strategy (tissue types and developmental stage) was adapted from the published transcriptome study by [28,31]. No RNA-seq data were generated for tissue-specific samples in this study. For each tissue type, three biological replicates were harvested, with each replicate representing an independent individual. Samples were immediately frozen in liquid nitrogen and stored at −80 °C until RNA extraction.

For the dark-to-light transition experiment, four-week-old *A. argyi* seedlings were dark-adapted for 7 days and subsequently transferred to continuous white light (500 μmol·m^−2^·s^−1^). Leaf samples were harvested at 0, 1, 6, 12, and 48 h after light exposure (top 2–3 healthy leaves per sample), with three biological replicates per time point. A total of fifteen libraries were sequenced. The genome data for *A. argyi* were obtained from the previous research [28].

### 2.6. RT-qPCR and Statistical Analysis

Total RNA was isolated from leaf tissue using an Omega Total RNA Extraction Kit (Omega Bio-tek, Norcross, GA, USA). RNA quantity and purity (A260/A280) were assessed with a NanoDrop spectrophotometer, and structural integrity was confirmed via 1.0% agarose gel electrophoresis. Following first-strand cDNA synthesis with a commercial reverse transcription kit (ABclonal, Wuhan, China), RT-qPCR was performed with SYBR Green using *β-actin* as the internal reference; primer sequences appear in Table S1. β-actin was selected as the reference gene based on prior validation in *A. argyi* across multiple tissues and under MeJA treatment [36], and supported by its successful application in light-responsive expression studies in *A. argyi* [37] and in the closely related *A. annua* [25,38].

Expression levels were calculated using the 2^−ΔΔCt^ method. RT-qPCR was performed with three independent biological replicates. Data are presented as mean ± SD. Significant differences between each light-treated time point and the dark control were assessed by one-way ANOVA followed by Dunnett’s multiple comparisons test, with adjusted *p*-values < 0.05 considered statistically significant. Primer specificity was checked using TBtools-II software (v2.400) in Primer Check module against the *A. argyi* genome. For homeologs sharing high sequence identity, gene-specific primers could not be designed, and the genes were analyzed as a homeolog group. See Supplementary Table S12 for primer sequences and matched gene IDs. Single-peak melting curves were observed for all primer sets (Supplementary Figure S1).

### 2.7. Promoter Cis-Acting Elements Analysis

Thirty-five key *AarBBX* genes were selected based on transcriptome and RT-qPCR data. From the 52 genes assigned to K-means clusters 1, 3, 4, 5, 6, and 7 (representing distinct light-responsive expression modules), 35 key candidates were selected based on high transcript abundance (FPKM ≥ 3.0 at any time point), and/or significant differential expression (fold change ≥ 2.0 at any light-treated time point vs. 0 h dark control). This combined criterion captured both early rapid responders (1 h peak) and delayed light-responsive genes (6–48 h peak), ensuring representative coverage of the light signaling network.

To analyze upstream regulatory networks, the 2 kb genomic region flanking the start codon of each *AarBBX* gene was extracted. Functional *cis*-acting regulatory elements within these promoter regions were predicted using PlantCARE (http://bioinformatics.psb.ugent.be/webtools/plantcare/html (accessed on 11 February 2025)) and subsequently mapped using TBtools-II software (v2.400).

### 2.8. Subcellular Localization in Tobacco

Nine representative genes were selected from a pool of 35 prioritized candidates for the purpose of subcellular localization analysis. These 35 candidates were initially derived from a total of 52 light-responsive genes, identified through K-means clustering based on RT-qPCR expression patterns and promoter cis-element profiles. Due to the high similarity among several of these 35 genes, characterized by an amino acid sequence identity exceeding 90.0%, we selected nine representatives that span distinct phylogenetic subfamilies and expression clusters. This approach was taken to validate nuclear localization while avoiding redundant testing of nearly identical proteins. The assay was conducted across three independent agroinfiltration experiments, with representative images provided.

Subcellular localization was identified online using WoLFPSOR. The coding sequences (minus stop codons) of nine candidate genes were cloned into pHB-GFP fusion vectors, transformed into *Agrobacterium tumefaciens* GV3101, and infiltrated into *Nicotiana benthamiana* leaves alongside a nuclear marker (NLS-mKATE). After 48 h under low light, GFP fluorescence was imaged on a laser-scanning confocal microscope (Nikon-C2-ER, Japan). Empty pHB-GFP served as a negative control.

### 2.9. Yeast Two-Hybrid (Y2H) Assays

The *AarHY5* coding sequence was inserted into the pLexA vector (BD vector in the LexA system), while the nine candidate *AarBBX* sequences were cloned into pB42AD (AD vector). Both plasmids were co-transformed into EGY48 competent cells (bearing p8op-LacZ) according to the Weidi (YC1031S) protocol. Autoactivation tests were performed by co-transforming each AD-prey (nine candidate *AarBBXs*) with empty BD, and BD-AarHY5 with empty AD. These combinations also served as negative controls for the respective interaction assays. There is no blue coloration on SGR/-His/-Trp/-Ura medium supplemented with X-gal, BU salts, raffinose and galactose for any control, confirming the absence of autoactivation [39,40]. The assay was independently repeated three times with consistent results.

### 2.10. Heatmap Visualization

Gene expression patterns of *A. argyi* leaves during dark-to-light transitions were displayed as a heatmap using the Metware Cloud Platform (https://cloud.metware.cn (accessed on 21 October 2025 and 13 June 2026)).

## 3. Results

### 3.1. Identification and Protein Properties of AarBBX Genes in A. argyi

Using AtBBX protein sequences as queries, we identified 114 *AarBBX* genes, designated *AarBBX1*-*AarBBX114* according to chromosomal position. Coding sequence lengths ranged from 492 to 1371 bp, corresponding to proteins of 163–456 amino acids with molecular weights of 17,848–51,805 Da. Theoretical isoelectric points (pI) ranged from 3.85 to 8.10, with most proteins below pH 7.0, indicating a predominantly acidic character likely due to high acidic amino acid content. All family members had an instability index exceeding 40, indicating they are unstable proteins. GRAVY analysis revealed negative values across all members, indicating hydrophilicity (Table S2).

### 3.2. Phylogenetic Relationship of AarBBX Genes

**Figure 1:**
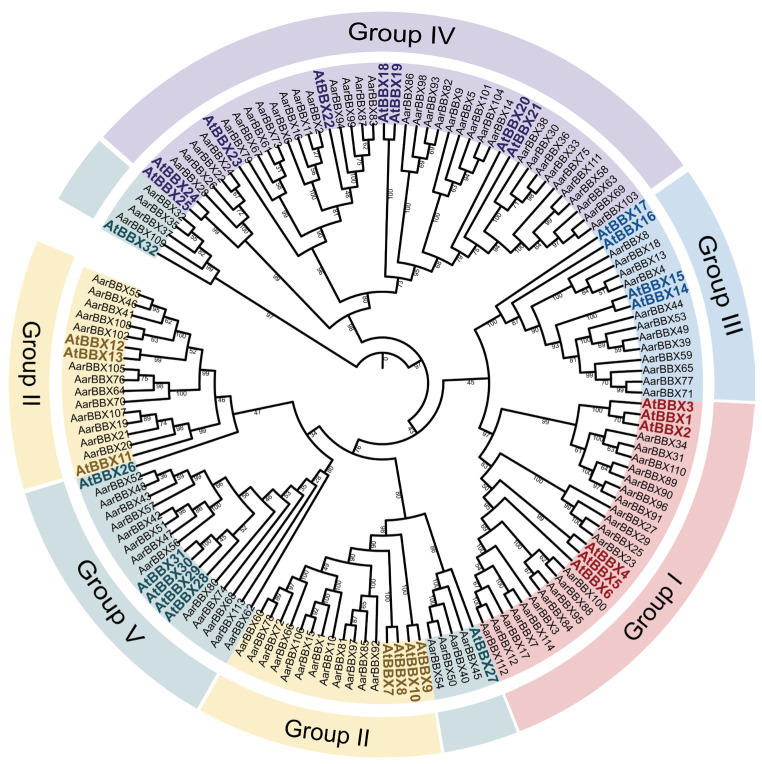
Phylogenetic evaluation of BBX proteins across *Arabidopsis thaliana* and *Artemisia argyi*. The ML tree was reconstructed using IQ-TREE under the JTT+R8 model with 1000 bootstrap replicates. Some *A. thaliana* subfamily members (e.g., Groups II and V) were not recovered as monophyletic clades, consistent with known topological sensitivity in deep-level BBX phylogeny.

We compared 114 AarBBX protein sequences with 32 previously characterized AtBBX proteins to determine evolutionary relationships. Based on the established classification of *A. thaliana* BBX proteins and their phylogenetic affinity in the combined ML tree, AarBBXs were categorized into five groups (I–V) (Figure 1). Group I contained 21 AarBBX and 6 AtBBX proteins (AtBBX1–6); Group II, 25 AarBBX and 7 AtBBX proteins (AtBBX7–13); Group III, 12 AarBBX and 4 AtBBX proteins (AtBBX14–17); Group IV, 35 AarBBX and 8 AtBBX proteins (AtBBX18–25); and Group V, 21 AarBBX and 7 AtBBX proteins (AtBBX26–32). Notably, phylogenetic analysis revealed that AarBBX members from *A. argyi* and AtBBX Group II/Group V members did not form independent monophyletic clades. Therefore, based on their clustering relationships with AtBBX reference sequences, *A. argyi* sequences that grouped with AtBBX Group II/Group V members were respectively designated as AarBBX Group II/Group V members in this study.

### 3.3. Gene Structure, Conserved Domains, and Conserved Motifs of AarBBXs

**Figure 2:**
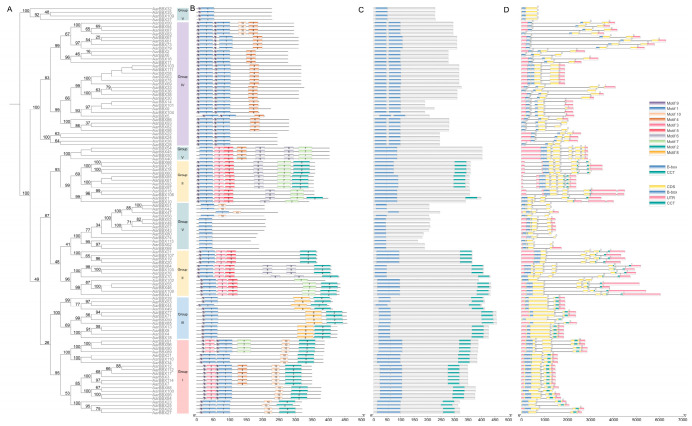
Phylogenetic relationships, motif, domain, and gene structure of AarBBX proteins. (**A**) The maximum likelihood method was employed to construct the rerooted phylogenetic tree. (**B**) Conserved motif clusters were visualized. Each motif is depicted in a distinct colored box. The scale at the bottom indicates the length of the protein. (**C**) The conserved B-box domains and CCT domain were identified using the CDD online tool and are displayed with blue and green boxes. (**D**) The gene structure for the putative *BBX* genes was analyzed, with yellow boxes, pink boxes, black lines, blue boxes, and green boxes denoting CDS, untranslated region (UTR), intron, B-box domain, and CCT domain, respectively.

Structural profiles, conserved domains, and motif compositions remained highly uniform within each distinct *AarBBX* subfamily (Figure 2). A MEME algorithmic search identified 10 distinct conserved motifs across the family. Motif 1 served as a universal signature sequence present in every family member, whereas Motif 2 was restricted entirely to Groups I, II, and III. Individual subfamilies displayed unique motif arrangements: Group I members were defined by a combination of Motifs 1, 2, 9, and 10; Group II featured Motifs 1, 2, 3, 5, and 9 (with Motifs 6 and 7 occurring in isolated genes); Group III was characterized by Motifs 1, 2, 8, and 9; and Group IV consisted primarily of Motifs 1 and 9, with sporadic occurrences of Motifs 4 and 10.

At the protein level, all AarBBXs carried either one or two N-terminal B-box domains. C-terminal CCT domains were standard across most subfamilies, appearing consistently in all sections except Groups IV and V. Mapping showed that Motifs 1 and 3 encoded these characteristic B-box structures, functioning either independently or in tandem. The structural absence of certain domains in a small number of subfamily members may point to local sequence gaps in the current genome assembly rather than evolutionary loss.

Exon-intron analysis revealed that intron counts across the 114 *AarBBX* genes ranged from 0 to 4. Specifically, 5 genes were intronless, 47 possessed a single intron, 33 contained two, 10 contained three, and 19 possessed four introns. Untranslated regions (UTRs) flanked the coding sequences of nearly all family members. Intron density was largely conserved within individual clades; Group I sequences typically contained 1–2 introns, whereas Group II genes were more complex, generally harboring 3–4 introns (with *AarBBX102* being the sole exception with 2).

### 3.4. Chromosomal Distribution, Duplication, and Synteny of AarBBXs

**Figure 3:**
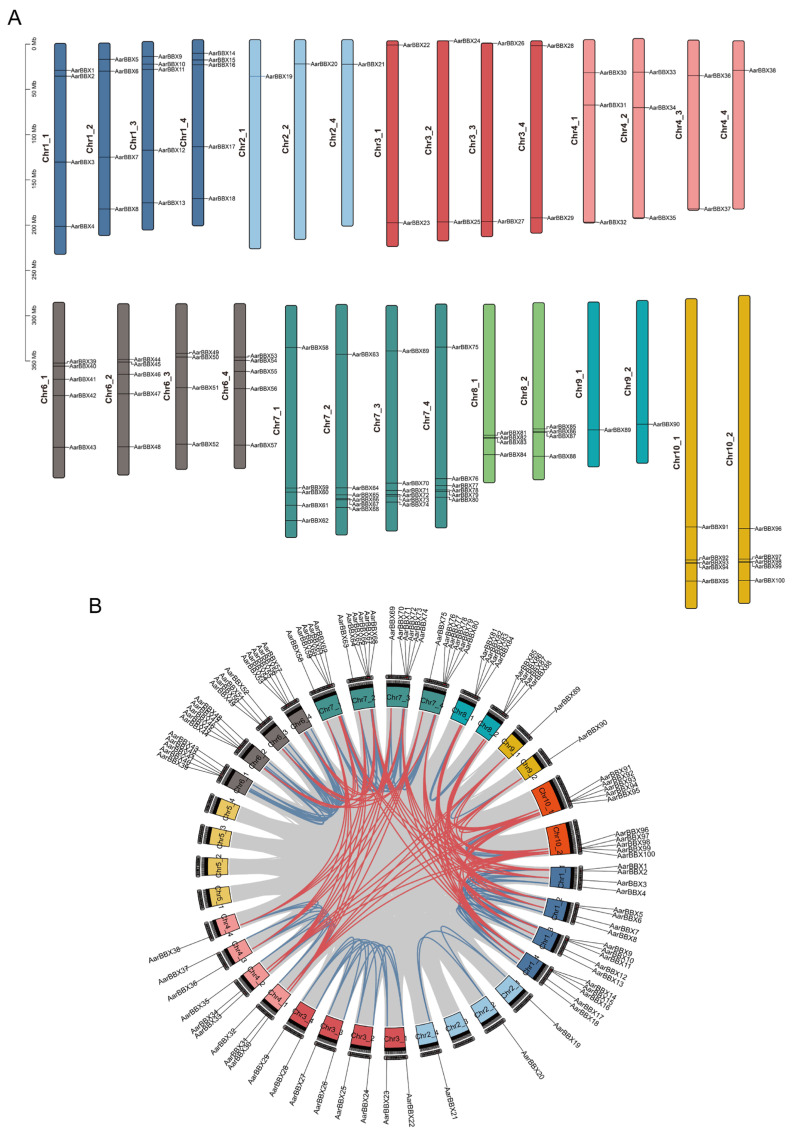
Chromosomal distribution and collinearity analysis of *AarBBXs* in *A. argyi*. (**A**) Chromosomal distribution of *AarBBXs*. The scale bar on the left represents the chromosome length (Mb). (**B**) Collinearity analysis of *AarBBXs*. The different color blocks denoted the segments of *A. argyi* chromosomes. The gray lines in the background represent collinear blocks within the genome of *A. argyi*, while the red lines indicate syntenic *AarBBX* gene pairs in different homologous groups, and the blue lines indicate syntenic *AarBBX* gene pairs in monoploid chromosomes of homologous groups. Homologous chromosomes are populated with the same color. ‘Chr’ means the chromosome.

The *A. argyi* genome is organized into 34 chromosomes spanning 10 distinct homologous sets: 7 sets contain 4 monoploid chromosomes, and 3 sets consist of pairs [28]. Out of the 114 identified family members, 100 were physically mapped across 29 chromosomes, displaying an uneven genomic distribution of 1 to 5 loci per chromosome (Figure 3A). Discrete gene clusters emerged on the homologs of Chr8 and Chr10, each anchoring two genes, while the remaining loci were distributed widely across the genome. Due to unresolved sequence scaffolds, 14 genes could not be assigned to specific chromosomal locations.

Collinearity analysis indicated that 93.0% (106/114) of the *AarBBX* genes arose through coordinated duplication events, while only 7.0% (8/114) lacked clear collinearity (Table S3). The identification of 237 syntenic gene pairs indicated that whole-genome duplications (WGD) or large-scale segmental duplications drove the expansion of this family (Figure 3B, Table S4).

**Figure 4:**
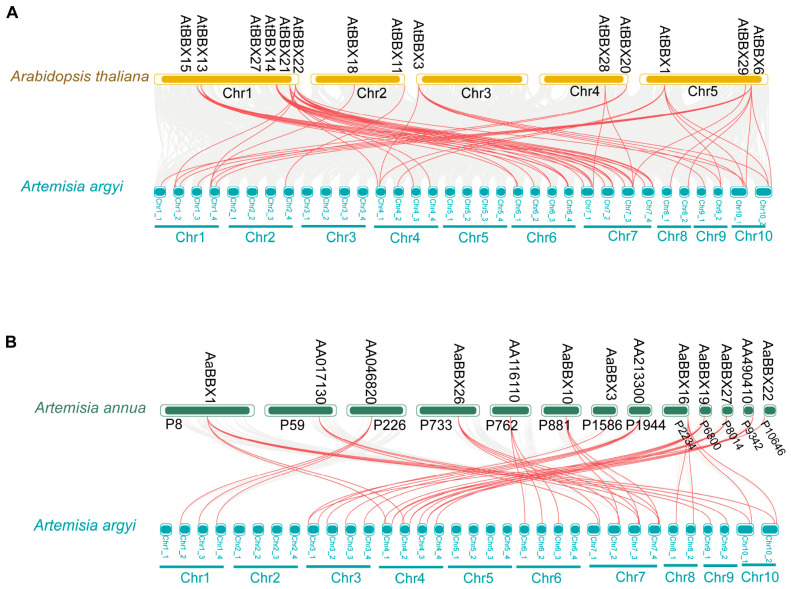
Syntenic relationships of *BBXs* between *Arabidopsis thaliana*, *Artemisia annua*, and *Artemisia argyi.* Collinear blocks are indicated by gray lines within the genomes of *A. thaliana* and *A. argyi* (**A**), *A. annua* and *A. argyi* (**B**), with syntenic pairs of *BBX* genes shown by red lines.

Cross-species synteny mapping against *Arabidopsis thaliana* revealed 56 collinear gene pairs (Figure 4, Table S5). Six *Arabidopsis* loci clustered on chromosome 1 (*AtBBX15/13/27/14/21/22*) showed direct synteny with 31 *AarBBX* genes. On chromosome 2, *AtBBX18* and *AtBBX11* matched *AarBBX14* and *AarBBX21*, respectively. On chromosome 3, *AtBBX3* corresponded to five *AarBBX* loci, while chromosome 4 loci *AtBBX20* and *AtBBX28* matched four *AarBBX* genes. Finally, chromosome 5 loci *AtBBX1*, *AtBBX6*, and *AtBBX29* exhibited synteny with 14 *AarBBX* genes. Synteny analysis between *A. argyi* and *A. annua* identified 42 collinear gene pairs, with single *A. annua BBX* genes frequently matching multiple homologs in *A. argyi* (Figure 4, Table S6). These syntenic networks highlighted a core evolutionary framework shared across these species, alongside extensive, lineage-specific gene expansion in *A. argyi*.

### 3.5. Light-Responsive Expression and Tissue Specificity of AarBBXs

The plant BBX family is central to modulating environmental stress responses, including cold tolerance, drought resistance, salinity adjustments, hormonal crosstalk, and light signaling [41,42,43,44]. To determine how light conditions affect *AarBBX* expression, transcriptomic profiling and RT-qPCR assays were performed on leaf tissue collected at 0, 1, 6, 12, and 48 h following a shift from dark to light.

**Figure 5:**
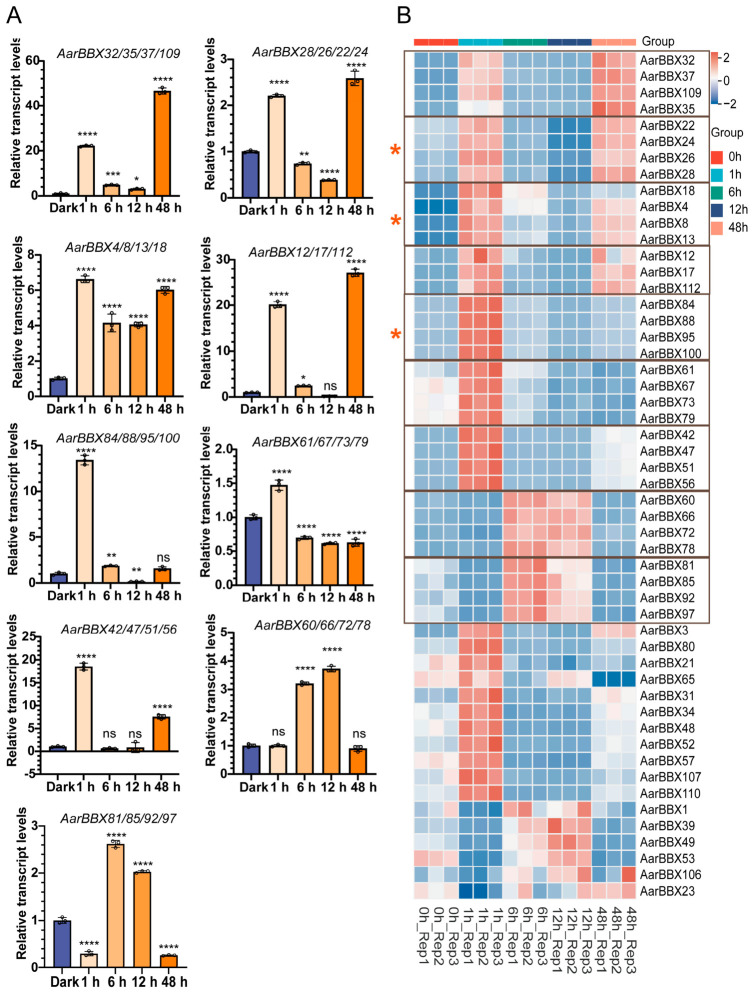
Gene expression profile and RT-qPCR analysis of core *AarBBXs* in response to light in *Artemisia argyi*. (**A**) The expression levels of key *AarBBXs* under dark-to-light conversion were analyzed by RT-qPCR. The samples placed in the dark were used as a control. Data represent the means ± SD of three biological replicates. Significant differences vs. dark control by one-way ANOVA with Dunnett’s test (* *p* < 0.05; ** *p* < 0.01; *** *p* < 0.001; **** *p* < 0.0001). ns, not significant (*p* > 0.05). Each circle represents an individual biological replicate (n = 3). (**B**) Heat map analysis of light-responsive *AarBBX* genes in cluster 1, cluster 3–7 from K-means clustering analysis. Genes with the strongest induction are marked with red asterisks.

Of the 114 family members, 112 were represented in the RNA-seq dataset, while two (*AarBBX101* and *AarBBX111*) fell below detection limits. K-means clustering separated 52 *AarBBX* genes that displayed marked upregulation post-exposure across clusters 1 and 3–7 (Figure 5B, Supplementary Figure S2, Table S7). From this light-responsive cohort, 35 high-expression candidate genes were prioritized for RT-qPCR validation (Figure 5A). Due to the allopolyploid nature of *A. argyi* and high sequence identity among duplicated genes, closely related paralogs were analyzed in groups because standard RT-qPCR primers could not distinguish between individual homeologs. For homeolog groups where primers matched multiple genes, the RT-qPCR data represented the combined expression of the matched group. This reflected the overall transcriptional response of these highly similar genes, but did not distinguish the contribution of individual homeologs.

The expression patterns determined by RT-qPCR were largely congruent with the transcriptome data: *AarBBX28/26/22/24* homeolog group, *AarBBX32/35/37/109* homeolog group, *AarBBX4/8/13/18* homeolog group, *AarBBX12/17/112* homeolog group, *AarBBX84/88/95/100* homeolog group, *AarBBX61/67/73/79* homeolog group, and *AarBBX42/47/51/56* homeolog group showed rapid upregulation within 1 h, decreased expression by 6 h, and then increased again by 48 h. Representative genes, including *AarBBX28/26/22/24* homeolog group, exhibited relative expression levels of 2.21-, 0.74-, 0.39-, and 2.58-fold at 1, 6, 12, and 48 h compared to 0 h after the dark-to-light transition, respectively. In contrast, *AarBBX60/66/72/78* homeolog group and *AarBBX81/85/92/97* homeolog group showed a delayed response, with significant upregulation at 6 and 12 h. Representative genes, including *AarBBX60/66/72/78* homeolog group, exhibited relative expression levels of 1.00-, 3.20-, 3.72-, and 0.90-fold at 1, 6, 12, and 48 h compared to 0 h after the dark-to-light transition, respectively. Both transcriptome and RT-qPCR data consistently showed that these genes responded positively to light treatment, suggesting their promising important roles in modulating light-dependent biosynthesis of medicinal metabolites, pending direct metabolic and pathway correlation evidence in *A. argyi*.

**Figure 6:**
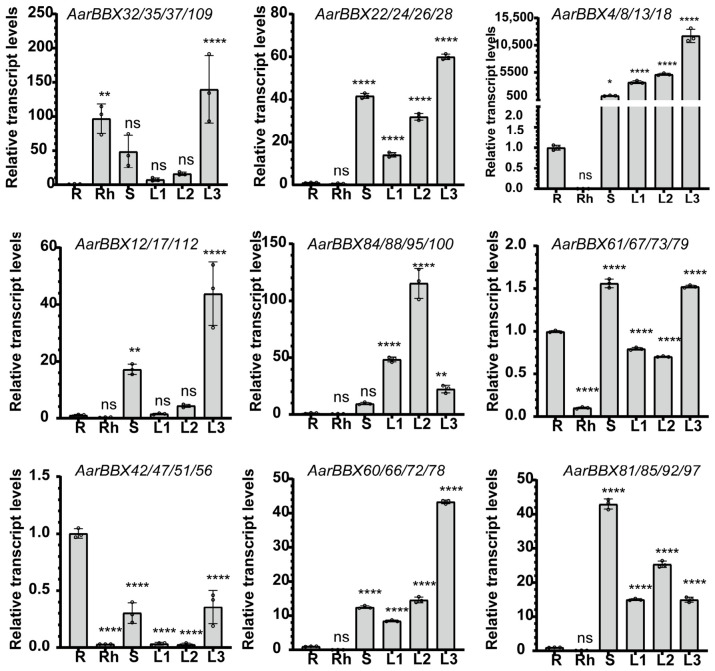
RT-qPCR analysis of relative expression patterns of core *AarBBX* genes in different tissues in *A. argyi*. R, root. Rh, rhizome. S, stem. L1, 5 days of leaf buds; L2, Leaf 2 representing 15 days of young leaves; L3, Leaf 3 representing 30 days of mature leaves. The samples in the root were used as a control. Data represent the means ± SD of three biological replicates. Significant differences vs. root control by one-way ANOVA with Dunnett’s test (* *p* < 0.05; ** *p* < 0.01; **** *p* < 0.0001). ns, not significant (*p* > 0.05). Each circle represents an individual biological replicate (n = 3).

Since medicinal compounds concentrate in *A. argyi* leaves, we examined tissue-specific expression of light-responsive candidates using RT-qPCR (Figure 6). Only *AarBBX42/47/51/56* homeolog group showed the highest expression in roots; all others displayed elevated leaf expression. *AarBBX28/26/22/24* homeolog group showed elevated levels in stems and mature leaves (L3). For example, *AarBBX28/26/22/24* homeolog group had tissue-specific expression, with transcripts in rhizomes (Rh), stems (S), and leaves at three developmental stages—leaf buds (L1, 5 days), young leaves (L2, 15 days), and mature leaves (L3, 30 days)—being 0.53, 41.75, 14.12, 31.88, and 60 times those in roots (R), respectively. The remaining gene homeolog groups, namely *AarBBX32/35/37/109*, *AarBBX4/8/13/18*, *AarBBX12/17/112*, *AarBBX84/88/95/100*, *AarBBX61/67/73/79*, *AarBBX60/66/72/78*, and *AarBBX81/85/92/97*, demonstrated the highest expression specifically in leaf tissues, consistent with the accumulation of medicinal constituents reported in *A. argyi*. However, these data nominated them as candidates for functional validation rather than establishing direct regulatory roles.

### 3.6. Promoter Cis-Acting Element Analysis of Candidate AarBBXs

**Figure 7:**
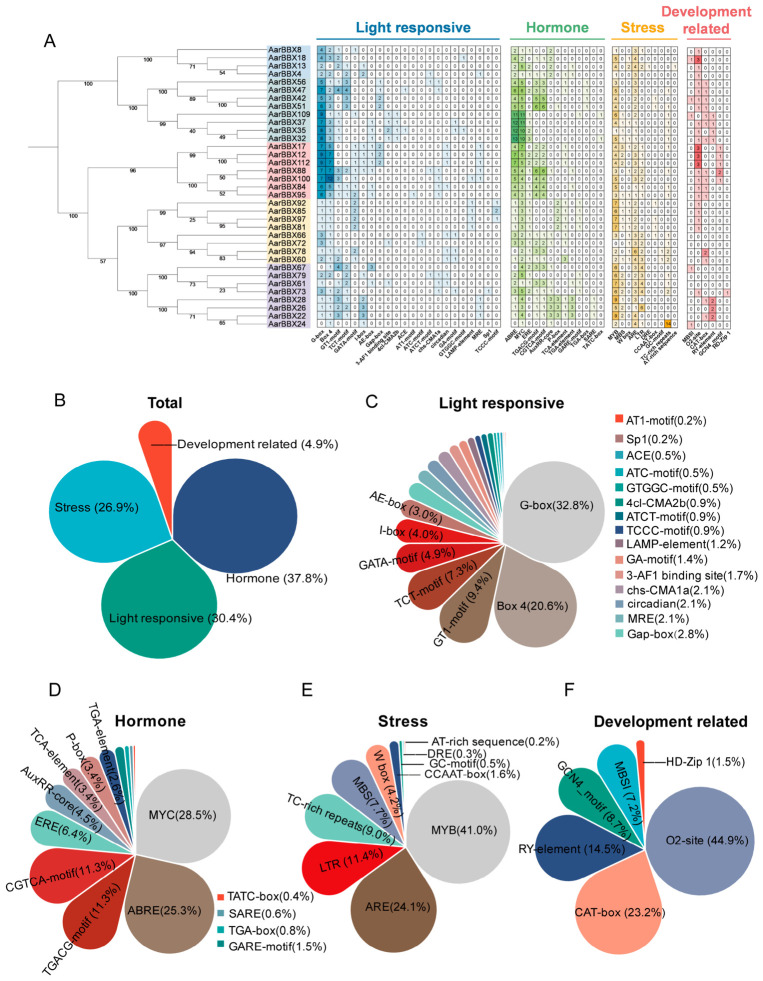
Analysis of cis-acting elements on the promoters of 35 candidate *AarBBX* genes in response to light. (**A**) The number of each *cis*-acting element present on the promoters of 35 candidate *AarBBX* genes is represented using varying shades of color and grid numbers (light responsive, blue; hormone, green; stress, orange; development related, red). The colored background of the gene name means different Group (blue, Group III; pink, Group I; yellow, Group II; purple, Group IV). (**B**–**F**) The pie charts show the overall representation of cis-acting elements related to total (**B**), light-responsive (**C**), hormone (**D**), stress (**E**) and development-related (**F**).

Thirty-five key light-responsive *AarBBX* genes were identified by K-means clustering analysis. Analysis of 2 kb of upstream sequences revealed *cis*-acting elements beyond the core promoter (CAAT-box, TATA-box), including widespread light-responsive, hormone-responsive, stress-adaptive, and development-related elements (Figure 7, Table S8). Among 1404 total elements, light-responsive comprised 30.4% (427), hormone-responsive 37.8% (530), stress-adaptive 26.9% (378), and development-related 4.9% (69) (Figure 7, Table S8).

The abundance of light-responsive elements aligns with BBX function in photomorphogenesis. The G-box (140 instances, 32.8%) dominated this category, serving as a binding site for bHLH and bZIP factors, including HY5, the core photomorphogenesis regulator (Figure 7). Promoter scanning revealed a heavy concentration of motifs sensitive to MeJA, ABA, auxin, and ethylene, pointing to a tight integration with stress-hormone signaling networks (Figure 7). Among these, MYC sites (151 counts, 28.5%) and ABRE features (134 counts, 25.3%) were the most abundant. Within the stress-adaptive category, MYB and MBS elements were predominant (184 counts, 48.7%), indicating that these transcription factors likely participate in potential MYB-mediated responsiveness to drought, osmotic imbalances, and salinity. Additionally, the presence of development-associated motifs, such as O2-sites, CAT-boxes, RY-elements, GCN4 motifs, and MBSI structures, suggests these loci also potentially respond to seed maturation, embryonic growth, and meristem maintenance. Notably, *AarBBX18*, *AarBBX42*, *AarBBX109*, *AarBBX67*, and *AarBBX24* contained MBSI elements (Figure 7A), common MYB binding sites associated with flavonoid pathways, indicating potential metabolic regulation. Collectively, these promoter features implicated the putative regulatory responsiveness of *AarBBX* genes to these stimuli (light signaling, abiotic stress adaptation, and hormone responses).

### 3.7. Subcellular Localization of Candidate AarBBX Proteins

All 114 AarBBX proteins were predicted to be nuclear-localized (Tables S2 and S9). Four proteins, namely AarBBX9, AarBBX14, AarBBX101, and AarBBX104, contained predicted Nuclear Export Signals (NES); AarBBX5 contained both NLS and NES; the remaining proteins contained NLS only.

**Figure 8:**
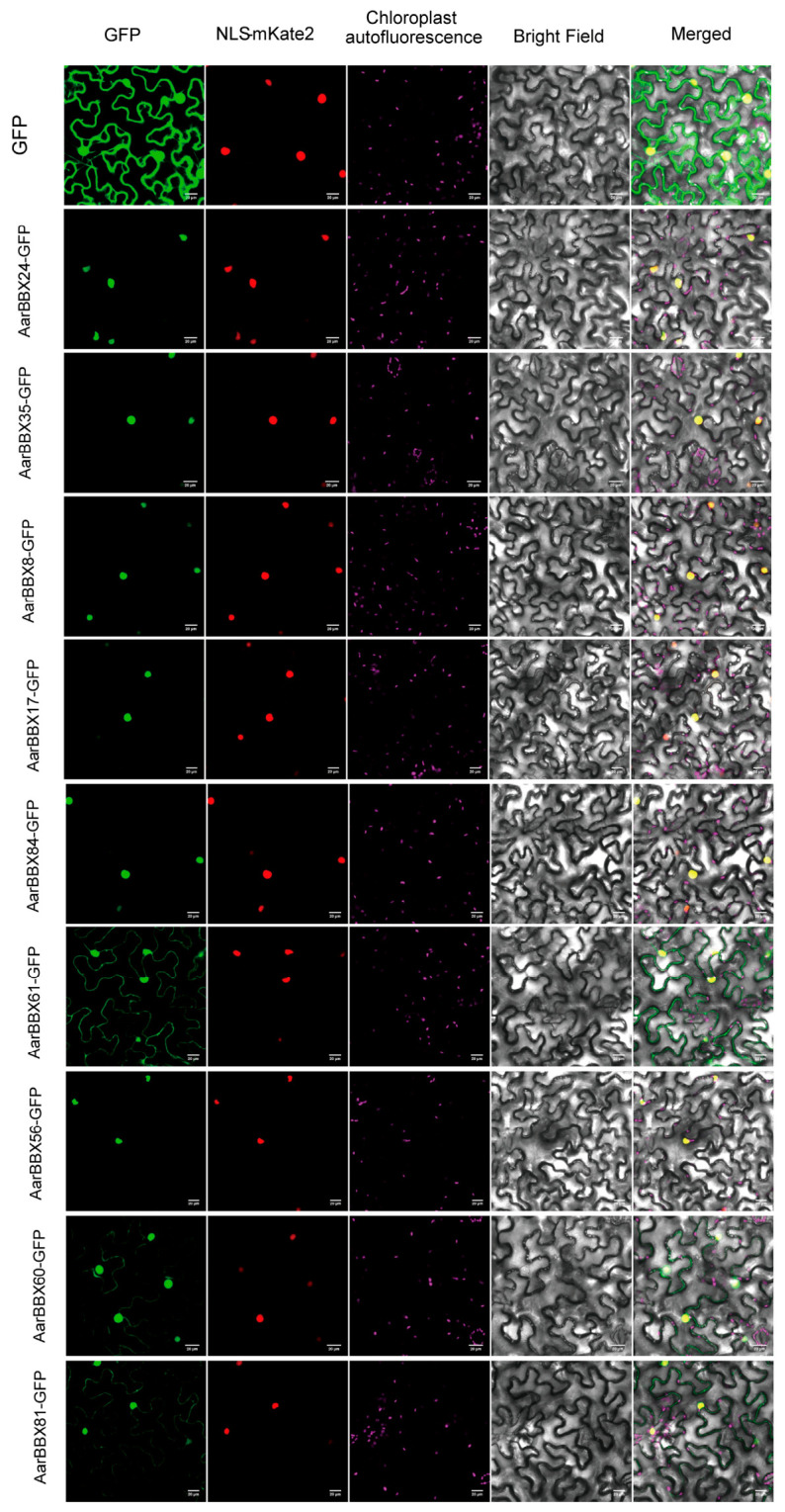
Subcellular localization analysis of GFP-fused AarBBX24/35/8/17/84/61/56/60/81 proteins. AarBBXs localization in *N. benthamiana* is shown; GFP, green fluorescence signal; Merged, superposition field. Scale bar (white) = 20 μm. Images are representative of three independent agroinfiltration experiments. GFP fluorescence (green) shows the AarBBX-GFP fusion protein; mKATE fluorescence (red) marks the nucleus via the NLS-mKATE marker; merged images (yellow) indicate nuclear localization.

Subcellular localization assays revealed nuclear localization for all nine representative *AarBBXs*. These nine genes were selected from 35 prioritized candidates (see Methods), given high sequence similarity among several paralogs. For example, due to the high sequence similarity among *AarBBX22/24/26/28*, RT-qPCR and proteomics cannot distinguish these homologs. We therefore selected AarBBX24 as a representative member of this quartet for subsequent validation experiments. Similarly, all nine light-responsive genes (*AarBBX24/35/8/17/84/61/56/60/81*) were cloned into pHB-GFP vectors and transiently expressed in *Nicotiana benthamiana* leaves. AarBBX24-GFP, AarBBX35-GFP, AarBBX8-GFP, AarBBX17-GFP, AarBBX84-GFP, AarBBX56-GFP, AarBBX60-GFP, and AarBBX81-GFP showed strong nuclear accumulation (Figure 8). AarBBX61-GFP localized to the nucleus with a weak cytoplasmic signal. The empty vector control (pHB-GFP) displayed intense, diffuse cytoplasmic and nuclear fluorescence. Results were consistent across three independent biological replicates (representative images are shown in Figure 8).

### 3.8. AarBBX-HY5 Protein Interactions

**Figure 9:**
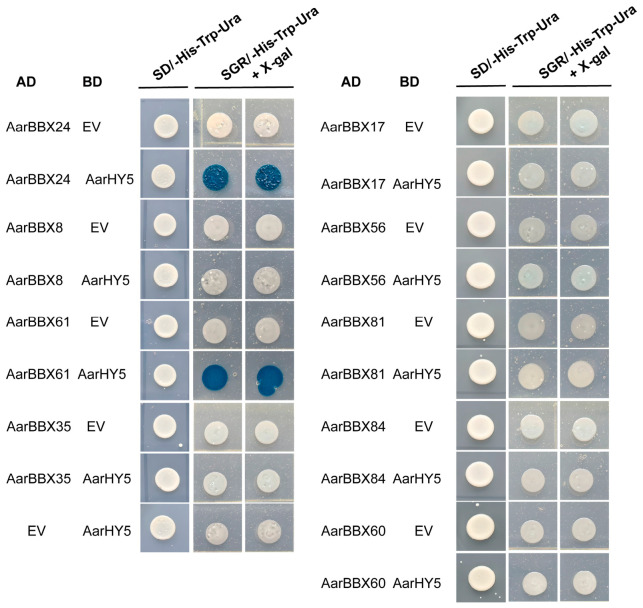
Yeast two-hybrid assays suggested a possible interaction of AarBBX24 and AarBBX61 with AarHY5 in yeast. Yeast two-hybrid (Y2H) assays for the interactions between AarBBX24/35/8/17/84/61/56/60/81 and AarHY5. Yeast grown on SD/-His/-Trp/-Ura plates means successful transformation. Yeast cells were spotted on SGR/-His/-Trp/-Ura plates supplemented with X-gal, BU salts, raffinose and galactose. Blue coloration indicates a positive interaction; white colonies indicate negative controls. EV, empty vector.

Transcription factors (TFs) control gene expression by binding to *cis*-elements in target promoters, thereby controlling the spatiotemporal patterns of downstream genes. BBX proteins mediate plant responses to multiple environmental stresses, including light, cold, drought, and salinity, as well as to phytohormones, and their expression is precisely tuned by upstream transcription factors [42,43]. In various plant species, BBX proteins also regulate photomorphogenesis and metabolite biosynthesis, largely depending on synergistic interactions with HY5, a core photomorphogenesis regulator [4,5,18,19,45]. Understanding AarBBX-HY5 interactions is essential for elucidating light-dependent metabolite regulation. We performed yeast two-hybrid (Y2H) assays with light-responsive AarBBX candidates (AarBBX24/35/8/17/84/61/56/60/81) and AarHY5. Only AarBBX24 and AarBBX61 directly interacted with AarHY5 (Figure 9).

### 3.9. Gene Expression Pattern Analysis of Group IV AarBBX Subfamily

Group IV BBX members regulate secondary metabolite biosynthesis across plant species [18,19,20,21,46]. In this study, transcriptome and qPCR data indicated that Group IV *AarBBXs* are highly expressed in tissues involved in secondary metabolism, suggesting a potential association. However, this inference is based solely on expression data, and direct functional evidence is still lacking.

We analyzed the 35 Group IV *AarBBX* genes using phylogenetic comparison with functionally characterized orthologs (*AtBBX18–25*, *SlBBX20*, *MdBBX20*, *CmBBX20*, *CsBBX22*, *PtrBBX23*, *PbBBX16*). Merging the RNA-seq datasets with RT-qPCR validation highlighted unique transcriptional trajectories for Group IV *AarBBX* members during dark-to-light shifts. Due to exceptionally high sequence identity among certain paralogs, some transcripts could only be evaluated collectively rather than as isolated genes.

*A. argyi* is an allotetraploid; several Group IV members were highly conserved homeologs that could not be distinguished by RT-qPCR. These genes were therefore analyzed and reported as homeolog groups (e.g., *AarBBX38/36/33* homeolog group), with primer specificity details provided in Supplementary Table S12 and Supplementary Figure S1.

**Figure 10:**
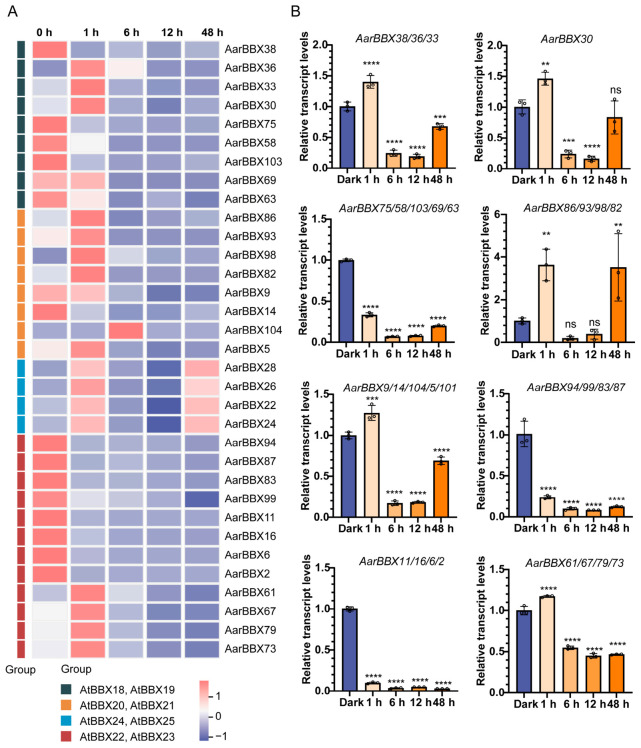
Identification and expression profiling of the BBX IV subgroup in *A. argyi.* (**A**) Heatmap analysis of *AarBBXs* in Group IV. (**B**) The relative expression levels of key *AarBBX* homeolog group were measured by RT-qPCR in different light conditions. The samples placed in the dark were used as a control. Data represent the means ± SD of three biological replicates. Significant differences vs. dark control by one-way ANOVA with Dunnett’s test (** *p* < 0.01; *** *p* < 0.001; **** *p* < 0.0001). ns, not significant (*p* > 0.05). Each circle represents an individual biological replicate (n = 3).

The validated loci fell into three main expression trends (Figure 5A,B and Figure 10A,B). The *AarBBX28/26/22/24* homeolog group cohort spiked sharply within 1 h of light exposure, dropped by the 6 h mark, and recovered again by 48 h. The homeolog groups comprising *AarBBX38/36/33* homeolog group, *AarBBX30*, *AarBBX86/93/98/82* homeolog group, *AarBBX9/14/104/5/101* homeolog group, and *AarBBX61/67/79/73* homeolog group all showed immediate early induction at 1 h, followed by a steady, continuous decline out to 48 h, ultimately dropping below baseline dark expression levels. Conversely, *AarBBX75/58/103/69/63* homeolog group, *AarBBX94/99/83/87* homeolog group, and *AarBBX11/16/6/2* homeolog group were downregulated by light exposure throughout the time course.

**Figure 11:**
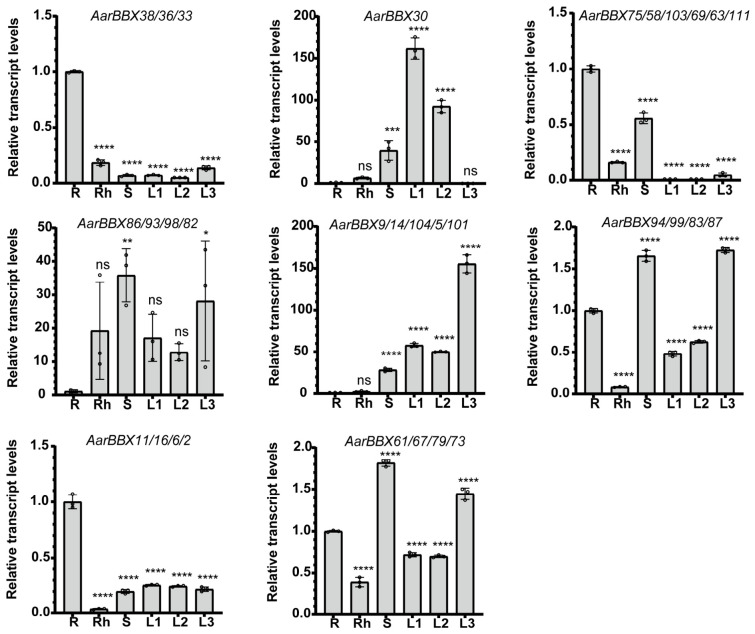
RT-qPCR analysis of relative expression patterns of BBX IV subgroup genes in different tissues in *A. argyi*. The relative expression levels of key *AarBBXs* were measured by RT-qPCR in different tissues. R, root. Rh, rhizome. S, stem. L1, 5 days of leaf buds; L2, Leaf 2 representing 15 days of young leaves; L3, Leaf 3 representing 30 days of mature leaves. Data represent the means ± SD of three biological replicates. Significant differences vs. root control by one-way ANOVA with Dunnett’s test (* *p* < 0.05; ** *p* < 0.01; *** *p* < 0.001; **** *p* < 0.0001). ns, not significant (*p* > 0.05). Each circle represents an individual biological replicate (n = 3).

To investigate the tissue-specific expression patterns of *AarBBX* genes, we performed RT-qPCR analysis in roots, rhizomes, stems, and leaves. Tissue-specific expression analysis across developmental leaf stages revealed functional specialization (Figure 6 and Figure 11). *AarBBX28/26/22/24* homeolog group accumulated in stems and leaves, with maximum transcript levels in mature leaves (L3), consistent with roles in leaf-specific metabolite synthesis. Homeolog groups *AarBBX38/36/33*, *AarBBX75/58/103/69/63/111*, and *AarBBX11/16/6/2* concentrated in roots. *AarBBX30* and *AarBBX9/14/104/5/101* showed elevated expression in leaves, with *AarBBX30* predominantly detected in early developmental stages (L1 and L2), whereas *AarBBX9/14/104/5/101* homeolog group was mainly expressed in mature leaves (L3). Homeolog groups *AarBBX94/99/83/87* and *AarBBX61/67/79/73* accumulated in stems (S) and mature leaves (L3). *AarBBX86/93/98/82* homeolog group exhibited minimal expression in roots, with relatively uniform transcript levels across other tissues. These patterns suggested that light-responsive Group IV members with high gene expression in leaves, particularly Homeolog groups *AarBBX28/26/22/24*, *AarBBX30*, *AarBBX9/14/104/5/101*, *AarBBX94/99/83/87*, and *AarBBX61/67/79/73* were nominated as candidates for future investigation into their potential relationship with medicinal constituent biosynthesis, pending metabolite profiling and genetic validation.

## 4. Discussion

### 4.1. WGD-Driven Expansion of the AraBBX Gene Family

We identified 114 *AarBBX* genes, substantially more than those of its diploid relative, *A. annua* (27 members), or the model plant, *A. thaliana* (32 members). This expansion reflects *A. argyi*’s complex evolutionary history. As an allotetraploid (2n = 34) with an 8.03 Gb genome, *A. argyi* experienced a minimum of three whole-genome duplication (WGD) events: *A. argyi* experienced at least three whole-genome duplication (WGD) events: the Asteraceae-shared WGT-1 (~62.9 Mya) and a recent *A. argyi*-specific WGD (~2.2–3.3 Mya) [28,47]. Among 114 *AarBBX* genes, only 7.0% (8/114) lacked collinear relationships; 93.0% (106/114) showed evident intraspecific collinearity. We identified 237 collinear gene pairs within the family, indicating expansion through ancient WGD or segmental duplication (Figure 3B, Tables S3 and S4). This pattern parallels the expanded MYB family in *A. argyi* (227 members) [31]. It remains unclear whether all 114 members are functionally retained or if a subset has undergone pseudogenization or subfunctionalization events. Consequently, the term ‘expansion’ is used here strictly in a numerical context, referring to an increased gene inventory rather than suggesting adaptive radiation or functional diversification of the BBX family in *A. argyi*.

The genetic redundancy resulting from whole-genome polyploidization events provided the evolutionary raw material necessary for functional diversification and expression specialization within this family. This expansion was further driven by uneven physical distribution across chromosomes, which generated localized gene clusters (Figure 3). Ultimately, this WGD-mediated duplication expanded the plant’s transcriptional regulatory network and likely provided the genetic foundation for *A. argyi*’s environmental adaptability and rich secondary metabolism.

Due to the allotetraploid nature of *A. argyi*, many homeologous genes retain highly similar sequences. It is difficult to distinguish individual homeologs by RT-qPCR. The grouped expression data therefore reflect the combined activity of the matched homeolog set. This approach is commonly used in polyploid species such as cotton and wheat [48,49]. Future work using homeolog-specific markers will be needed to study the function of individual copies.

### 4.2. Domain Conservation and Functional Differentiation Among BBX Subgroups in A. argyi

Following the established classification frameworks for *A. thaliana*, the *AarBBX* family was partitioned into five major subgroups (I–V) by integrating domain architectures with phylogenetic relationships. Although recent structural evaluations suggest reclassifying AtBBX26 and AtBBX27 into Group II because of previously overlooked B-box configurations and degenerate CCT domains [16,50,51], this study maintains the classic *Arabidopsis* taxonomic arrangement (Figure 1). Minor topological variations, such as the unique branching arrangements of Groups II and V, are attributable to our reliance on Maximum Likelihood phylogenetic reconstruction rather than the traditional Neighbor-Joining approach (Figure 1).

Despite sharing well-conserved core domains, distinct BBX subgroups display substantial functional divergence, which directly impacts how they modulate light signaling cascades and secondary metabolic pathways. Group IV members are highly specialized in metabolic regulation, directing the synthesis of anthocyanins, phenolic acids, flavonoids, carotenoids, and artemisinin [18,19,25,52,53]. Interestingly, AaBBX22 and its *Arabidopsis* counterpart AtBBX24 (STO) exhibit a functional inversion despite their structural similarities: AtBBX24 suppresses photomorphogenesis and downregulates anthocyanin synthesis [54], whereas AaBBX22 acts as a positive regulator of artemisinin accumulation [25]. This functional contrast suggests that AarBBX proteins achieved specific pathway control through mutations in variable C-terminal regions or variations in downstream promoter binding affinities.

Notably, knockouts or partial deletions of the *ADS* locus in *A. argyi* halt artemisinin synthesis entirely, creating a clear metabolic distinction from *A. annua*, which depends on *AaADS* for artemisinin assembly [28,29,30]. Nevertheless, the overarching BBX regulatory machinery seems conserved. This indicates that AarBBX proteins may have undergone target gene rewiring, shifting their regulatory focus from artemisinin synthesis toward terpenoid or flavonoid production in *A. argyi*. Such “regulatory factor retention with target gene switching” likely represents a common evolutionary strategy in polyploid secondary metabolism. It remains a high priority for future research to determine whether Group IV *AarBBX* members govern the production of characteristic *A. argyi* volatile oils and flavonoids via these exact rewiring mechanisms.

### 4.3. Promoter Cis-Elements of AarBBXs Form a Multi-Signal Regulatory Network

Promoter analysis demonstrated a high density of functional cis-elements spanning multiple regulatory networks: light perception (G-box, TCT-motif, Box4), hormonal signaling (ABRE, MYC, ERE), stress adaptation (MYB, MBS, W-box, ARE), and plant development (MBSI, O2-site, CAT-box). This distribution parallels that of *AaBBX* promoters in *A. annua* [25], positioning *BBX* genes as central integration hubs for environmental and developmental signals in *A. argyi*.

Light-responsive cis-elements were the most abundant, which correlates closely with the expression profiling observed during our dark-to-light transition experiments. G-box elements, the classic binding targets for the HY5 transcription factor, were enriched within the promoters of light-activated *AarBBX* genes. This pattern implicates these genes in the core “HY5-BBX-COP1” photomorphogenic signaling framework. Furthermore, a strong positive correlation between G-box copy number and the magnitude of transcript induction suggests that HY5 either directly drives *AarBBX* expression or coordinates with these factors to co-activate downstream targets. Within the Asteraceae family, light exposure stimulates both morphological de-etiolation and the targeted accumulation of defensive flavonoids and terpenoids in foliar tissues [19,25,52]. Previous studies have demonstrated that the “AaBBX21-AaHY5” module in *A. annua* exemplifies this coupling: during illumination, cytoplasmic AaCOP1 is sequestered, while nuclear AaHY5 and AaBBX21 synergistically activate artemisinin regulators (*AaGSW1, AaMYB108,* and *AaORA*); in darkness, AaCOP1 triggers AaHY5 and AaBBX21 degradation, suppressing artemisinin synthesis [19]. Since *A. argyi* leaves accumulate light-dependent flavonoids (centaureidin, eupatilin) and terpenoids (1,8-cineole, camphor, borneol) [37], the rapidly light-induced *AarBBX* genes with high leaf expression likely function as signal transducers coupling photosignals to secondary metabolic reprogramming.

Hormone-responsive elements dominated *AarBBX* promoters, particularly ABA- and MeJA-responsive elements (ABRE, MYC). In *A. annua*, *AaBBX5/6/8/15/22/23/24* respond strongly to MeJA and ABA, interacting with JAZ8 to relieve JA-mediated repression of artemisinin genes [25]. As *A. argyi*’s close relative, its BBX family likely participates in similar hormone cross-talk networks.

Stress-responsive elements (AREs, MBSs, TC-rich repeats) were abundant, reflecting *A. argyi*’s exposure to drought, salinity, and pathogens across its East Asian range. These findings align with functional behaviors documented in other species. For example, CmBBX19 bolsters drought tolerance in chrysanthemum [44], while AtBBX24/STO establishes salt tolerance in *Arabidopsis* [46]. This suggests that *AarBBX* genes serve a parallel role in environmental adaptation, directly reinforcing the high ecological resilience of *A. argyi*.

### 4.4. Light-Responsive Expression in Leaves Implicates AarBBX in Secondary Metabolite Regulation

Foliar tissues serve as the primary metabolic engines for synthesis of both volatile oils and therapeutic flavonoids within *A. argyi*. Integrated transcriptome data and the analysis of metabolite detection show that 1,8-cineole, borneol, camphor, and flavonoids (eupatilin, jaceosidin) accumulate progressively with leaf maturation [28]. Several identified *AarBBX* transcripts exhibit strong leaf-specific expression profiles that mirror these compound accumulation curves, marking them as probable upstream regulators of local specialized metabolic pathways.

Dark-to-light transition transcriptomes provided critical insights into dynamic regulation in *A. argyi*. K-means clustering identified *AarBBX* genes that showed rapid expression changes following illumination, with some upregulated quickly as potential light signal transducers. This dynamic responsiveness parallels the abundance of light-responsive elements in their promoters, confirming their active participation in light signaling.

In *A. annua*, AaBBX22 functions as a master controller of artemisinin biosynthesis, showing high expression in glandular secretory trichomes and directly activating *AaADS*, *AaCYP71AV1*, *AaDBR2*, and *AaALDH1* promoters in a light-dependent manner [25]. The “AaBBX21-AaHY5” module synergistically activates downstream artemisinin regulators (*AaGSW1, AaMYB108,* and *AaORA*) in response to light signals [19]. Although *A. argyi* lacks functional artemisinin synthase, it compensates with expanded MEP pathway genes and terpene synthase (TPS) families, plus tandem-duplicated borneol/camphor gene clusters (*BPPS*, *BDH*). Flavonoid biosynthesis genes, namely *CHS*, *F3’H*, and *FOMT*, similarly show substantial genome expansion [28,29,30].

Based on the interactions between AarHY5 and AarBBXs identified through yeast two-hybrid (Y2H) analysis, along with the high expression levels of these genes in leaves, we propose a working hypothesis: light-responsive AarBBX24 (or AarBBX61) may collaborate with AarHY5 to influence the expression of *TPS* and flavonoid biosynthetic genes. It remains to be determined whether this interaction occurs through direct promoter binding or transactivation.

Notably, cross-species synteny analysis identified *AarBBX22* and *AaBBX22* as ortholog pairs (Table S6). The quartet *AarBBX22/24/26/28* homeolog group, located on the four haploid Chr3 copies (Chr3.1–Chr3.4), shares high sequence identity and light-responsive expression (Figure 5, Table S7). A limitation of this study is that *AarBBX*22/24/26/28 share extremely high sequence similarity, making them indistinguishable by RT-qPCR. We cloned *AarBBX24* as a representative for subcellular localization and interaction assays. Subcellular localization and Y2H assays confirmed nuclear accumulation and the interaction with AarHY5 (Figure 8 and Figure 9), positioning AarBBX24 as potential metabolic regulators for future functional validation. Consequently, the localization and interaction patterns reported here reflect AarBBX24 specifically. Whether AarBBX22, AarBBX26, and AarBBX28 exhibit identical behavior remains to be determined by individual characterization of each homolog.

This study represents a primary screening effort aimed at identifying light-responsive members within the *AarBBX* gene family. The differential expression patterns reported here serve solely as a basis for candidate prioritization and do not constitute evidence of direct participation in flavonoid or terpenoid biosynthesis. Further experiments, metabolite profiling, target-promoter binding assays, and genetic validation are required to test whether these expression-responsive candidates are functionally linked to flavonoid/terpenoid accumulation in *A. argyi*. EMSA and dual-luciferase reporter assays, are needed to validate the binding of these BBX proteins to promoters of downstream metabolic genes, assess their transactivation activity, and evaluate their cooperative interactions with HY5. Critical needs include developing *A. argyi* genetic transformation systems for transgenic overexpression and CRISPR-based knockouts to definitively establish gene function. Together, this work establishes a foundation for exploring potential links between *AarBBX* genes and light-regulated secondary metabolism.

## 5. Conclusions

We identified 114 *AarBBX* genes and characterized their chromosomal locations, phylogenetic relationships, conserved domains, gene structures, promoter *cis*-elements, and light-responsive gene expression. Following *A. thaliana* classification, five subgroups emerged; while domain organization remained conserved, variable gene structures reflected functional specialization driven by whole-genome duplication. Expression profiling, RT-qPCR, subcellular localization, and yeast two-hybrid screening revealed key candidates, *AarBBX24* and *AarBBX61*, that show high expression in leaves and rapid light induction, suggesting potential roles in regulating flavonoid and terpenoid biosynthesis in *A. argyi*. Given that stable genetic transformation systems remain technically challenging and time-consuming for *A. argyi*, this provides an essential framework for selecting candidate genes for downstream functional validation. These findings identify light-responsive *AarBBX* genes that exhibit differential expression during dark-to-light transitions, providing a candidate set for future investigations into their potential relationship with light-mediated secondary metabolite accumulation and enhancements in pharmaceutical yield.

## Data Availability

The raw RNA-seq data for the dark-to-light transition experiment generated in this study have been deposited in the NCBI Sequence Read Archive (SRA) under BioProject accession number PRJNA1181633. The accession numbers for individual libraries are listed in Supplementary Table S11.

## Supplementary Materials

The following supporting information can be downloaded at https://www.mdpi.com/article/10.3390/plants15132003/s1: Table S1: List of primers used in this study; Table S2: Physicochemical Properties of AarBBX Protein in *Artemisia argyi*; Table S3: Modes of gene duplication of *AarBBX* genes; Table S4: 237 pairs of genes belonging to the *AarBBX* gene family were obtained on the basis of collinearity analysis; Table S5: 56 pairs of *BBX* genes between *Arabidopsis thaliana* and *Artemisia argyi* on the basis of synteny analysis; Table S6: 42 pairs of *BBX* genes between *Artemisia annua* and *Artemisia argyi* on the basis of synteny analysis; Table S7: FPKM and basic information of 52 key *AarBBX* on the basis of K-means clustering analysis from dark-to-light RNA-seq; Table S8: List of the *cis*-acting elements identified in this study; Table S9: Subcellular localization of *AarBBX* gene family; Table S10: FPKM and basic information of 33 key *AarBBX* in Group IV on the basis of K-means clustering analysis from dark-to-light RNA-seq. Table S11: Information and SRA accession numbers of RNA-seq libraries for the dark-to-light transition experiment. Table S12: Summary of RT-qPCR primer pairs and their specificity validation using TBtools in silico PCR against the *A. argyi* genome. Figure S1: Melting curve analysis of RT-qPCR primer pairs. Single-peak melting curves were observed for all primer sets, confirming the absence of non-specific amplification. X-axis, Temperature (°C); Y-axis, -Rn’ (negative derivative of normalized reporter). Figure S2: K-means clustering analysis of genes in response to light in *Artemisia argyi*. The numbers represented the numbers of DEGs in the clusters. Figure S3: Phylogenetic evaluation of *BBX* genes in Group IV across *A. argyi* and other species. The ML tree was reconstructed using IQ-TREE under the JTT+I+G4 model with 1000 bootstrap replicates using the TBtools-II (v2.400) based on BBX sequences from *A. argyi* genome and published data.
