# Supplementary material for: Genome-Wide Characterization and Light-Responsive Expression Patterns of B-Box Transcription Factors in Artemisia argyi
Source: Plants (Basel). 2026 Jun 28;15(13):2003. doi: 10.3390/plants15132003 (PMC13363855; doi:10.3390/plants15132003)
Supplement: Supplementary file 1 [file plants-15-02003-s001.zip › Supplementary Figures_revised.pptx]

## Slide 1
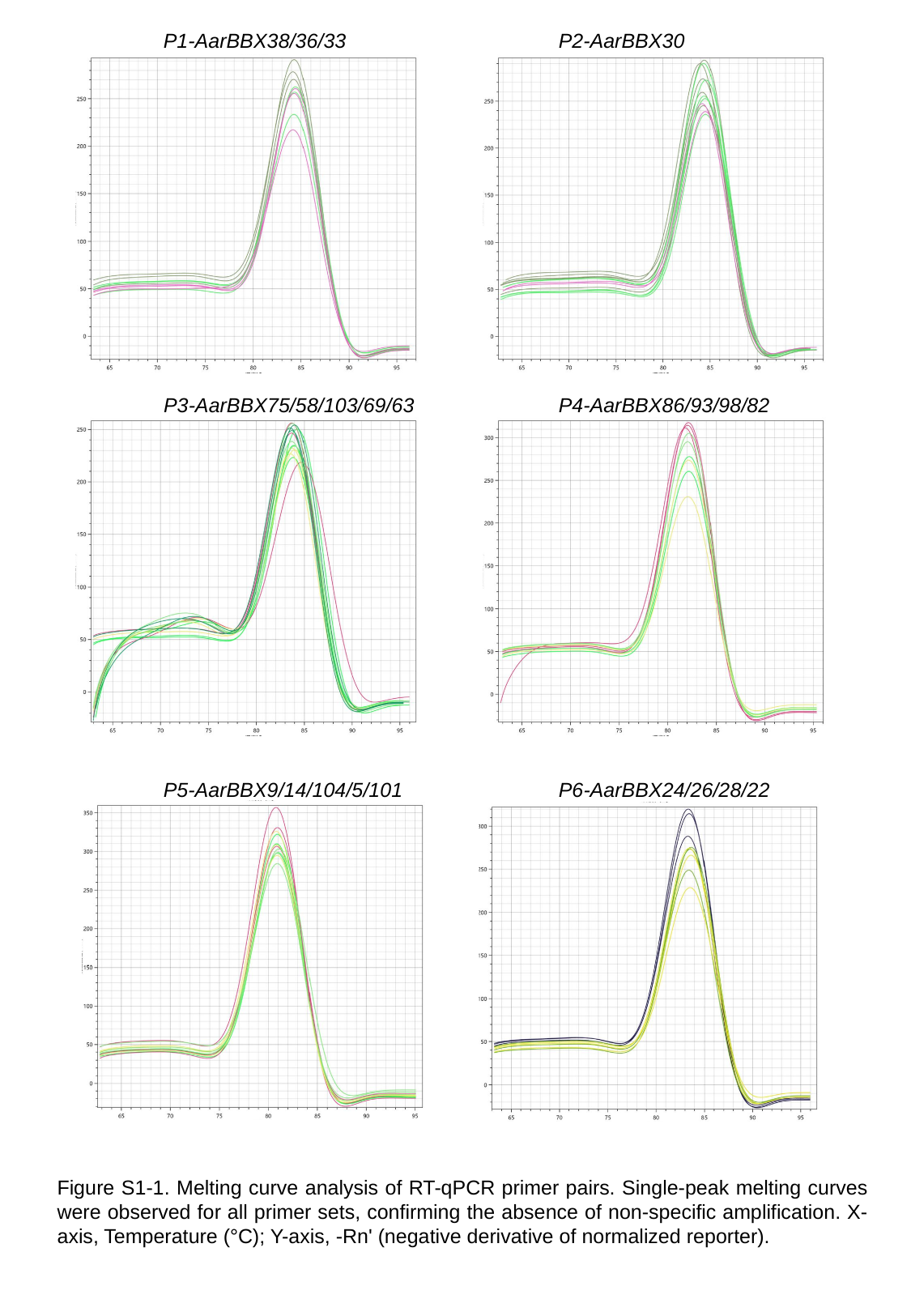

P1-AarBBX38/36/33
P2-AarBBX30
P3-AarBBX75/58/103/69/63
P4-AarBBX86/93/98/82
P5-AarBBX9/14/104/5/101
P6-AarBBX24/26/28/22
Figure S1-1. Melting curve analysis of RT-qPCR primer pairs. Single-peak melting curves were observed for all primer sets, confirming the absence of non-specific amplification. X-axis, Temperature (°C); Y-axis, -Rn' (negative derivative of normalized reporter).

## Slide 2
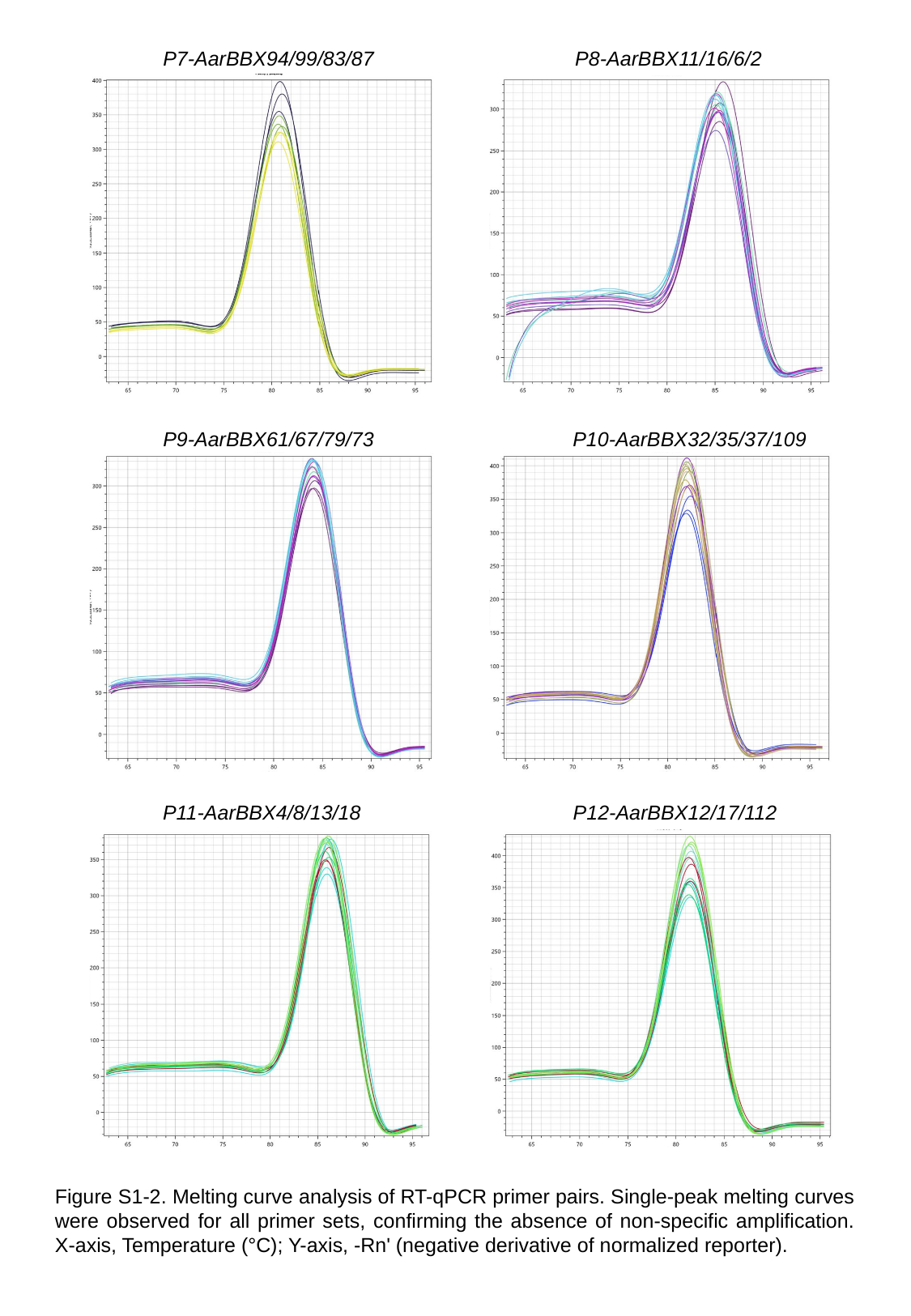

P7-AarBBX94/99/83/87
P8-AarBBX11/16/6/2
P9-AarBBX61/67/79/73
P10-AarBBX32/35/37/109
P11-AarBBX4/8/13/18
P12-AarBBX12/17/112
Figure S1-2. Melting curve analysis of RT-qPCR primer pairs. Single-peak melting curves were observed for all primer sets, confirming the absence of non-specific amplification. X-axis, Temperature (°C); Y-axis, -Rn' (negative derivative of normalized reporter).

## Slide 3
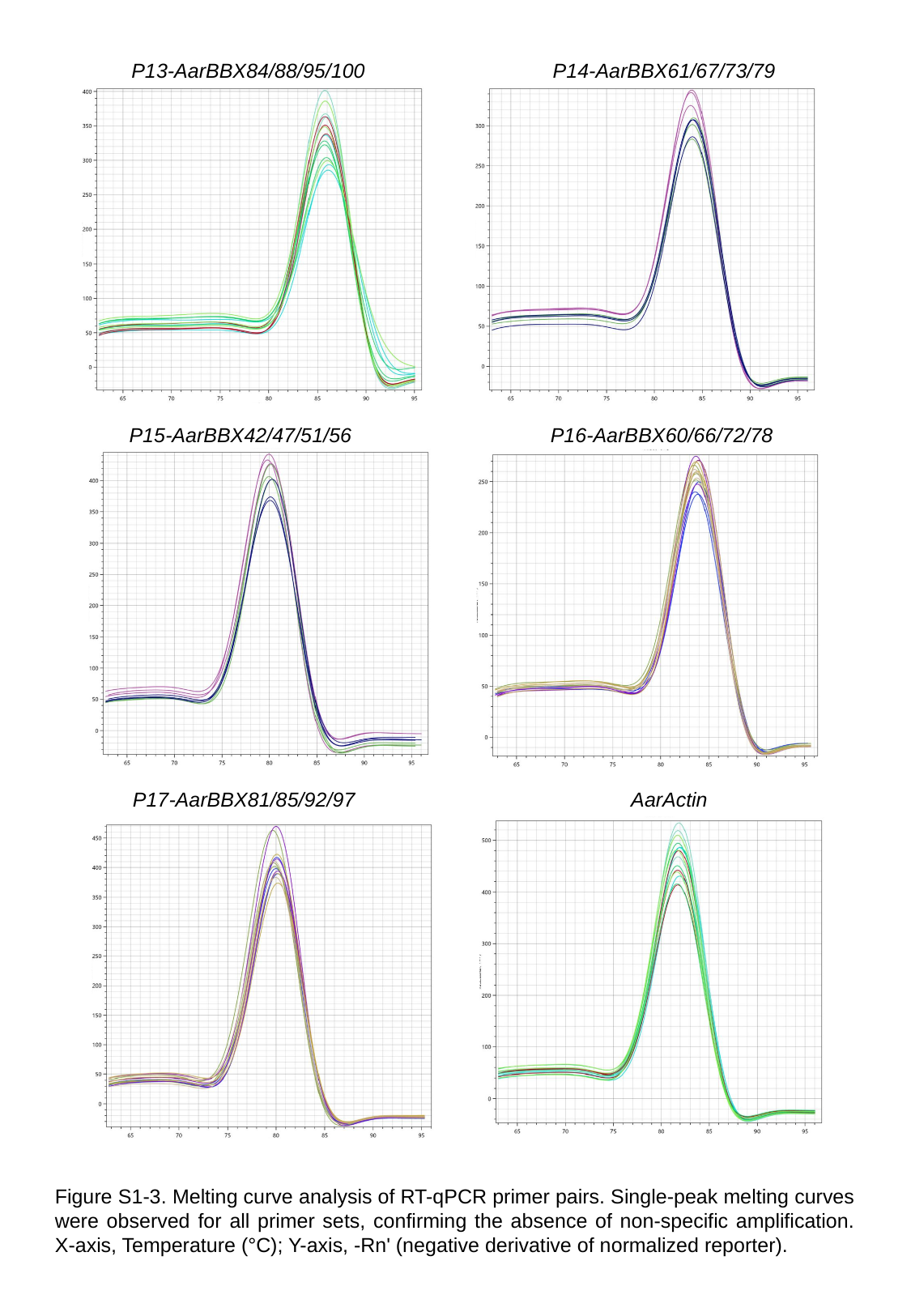

P13-AarBBX84/88/95/100
P14-AarBBX61/67/73/79
P15-AarBBX42/47/51/56
P16-AarBBX60/66/72/78
P17-AarBBX81/85/92/97
AarActin
Figure S1-3. Melting curve analysis of RT-qPCR primer pairs. Single-peak melting curves were observed for all primer sets, confirming the absence of non-specific amplification. X-axis, Temperature (°C); Y-axis, -Rn' (negative derivative of normalized reporter).

## Slide 4
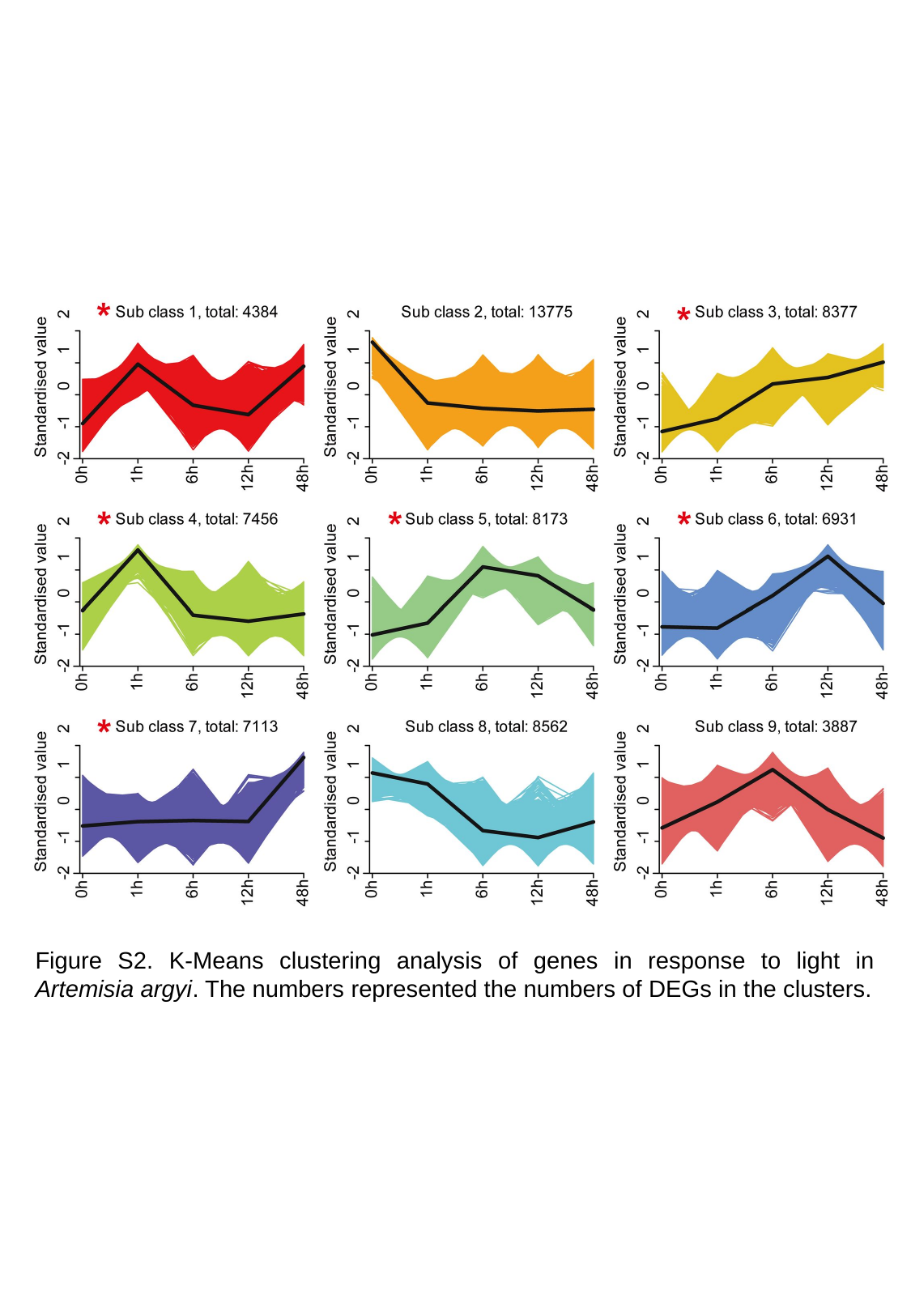

Figure S2. K-Means clustering analysis of genes in response to light in Artemisia argyi. The numbers represented the numbers of DEGs in the clusters.

## Slide 5
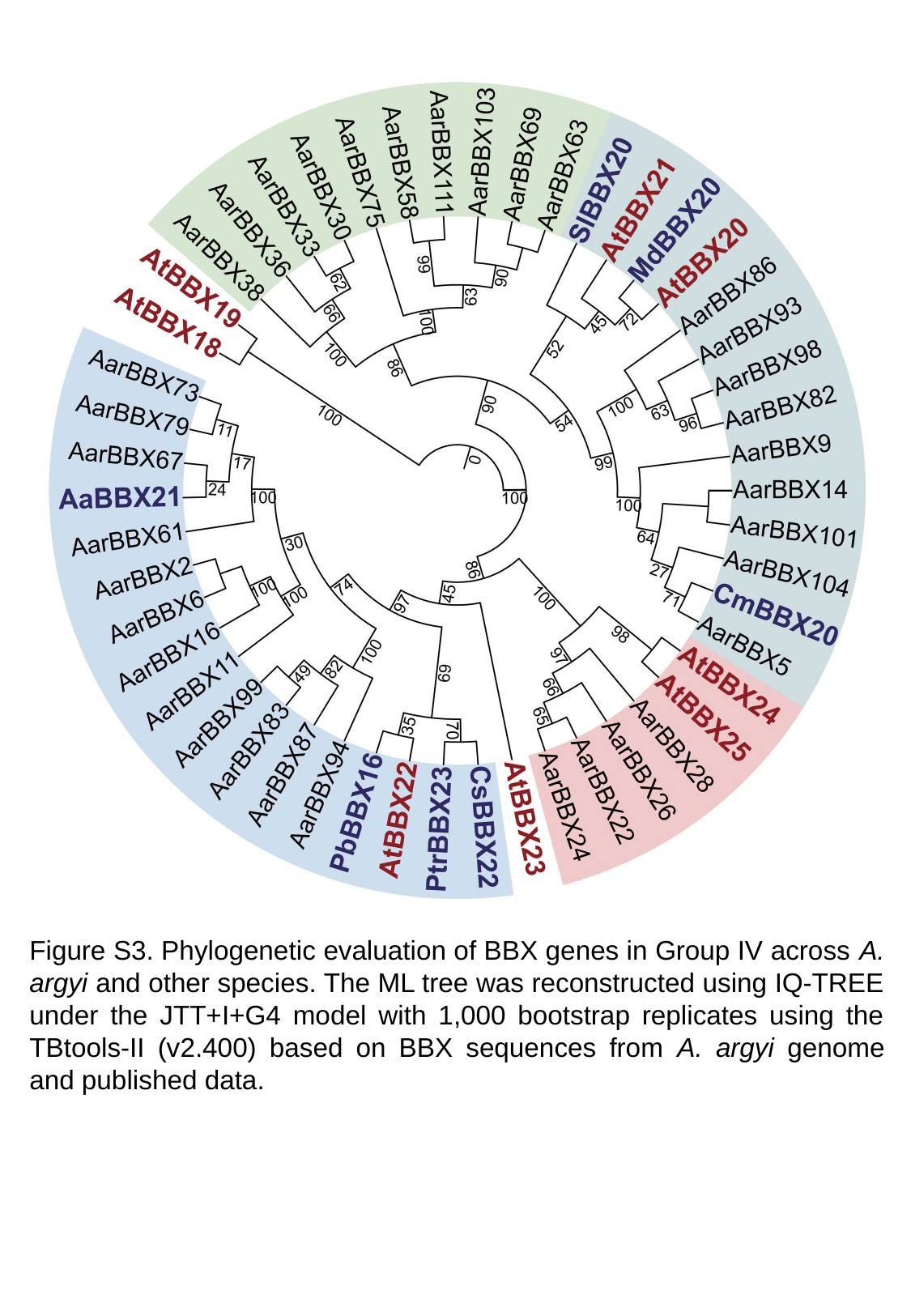

Figure S3. Phylogenetic evaluation of BBX genes in Group IV across A. argyi and other species. The ML tree was reconstructed using IQ-TREE under the JTT+I+G4 model with 1,000 bootstrap replicates using the TBtools-II (v2.400) based on BBX sequences from A. argyi genome and published data.
